# Dr. Clendinning’s Case of Hallucination and Epilepsy

**Published:** 1842-04-30

**Authors:** John Clendinning

**Affiliations:** Senior Physician to St. Marylebone Infirmary


*Hallucination and Epilepsy.* By John Clendinning, M. D., F. R. S., Senior Physician to St. Marylebone Infirmary.—There was a case amongst the discharges of the last week which had been about three weeks under treatment for mental hallucination, connected with fits, which I must notice. This woman was fifty-seven years of age, and had for nine years been subject to occasional fits of an epileptic character. The night before her admission she had one, and after her recovery from the fit her mind was affected; she said she had been bewitched by somebody in the workhouse; on every other subject her mind appeared to me quite clear, but on this subject she never hesitated; she seemed quite satisfied of the reality of her fancy. I listened to her story gravely, not attempting to dispute with her about it, as I knew I might add to her excitement materially by doing so, and that I should certainly not succeed in undeceiving her. On examining her person, I found the head hot, the carotids full, and resisting compression strongly; her manner and expression indicated excitement; she complained of headach. She was put on broth diet, and was cupped on the nape to eight ounces, and had a senna draught immediately. Cold was then applied to the head, and light antimonials were ordered. In a day or two the head was much relieved, and she told me that she had no trouble from the witch after the second day of treatment. On the fifth day, however, I cupped her again to eight ounces, and on the ninth day all trace of determination to the head was gone, and weakness only remained: her hallucination had disappeared some days earlier.

*Remarks.*—This witchcraft is a common fancy of this class of wrong-heads, or a common form of what is now called monomania. We have another example of the illusions up stairs at this moment: it is a case you have all seen in Alderton's ward, of a woman seventy-eight, admitted January 24th, with head symptoms, like those of the case just detailed. This old lady assures me that for a long time the spirit of some person that is dead has lain on her, and caused her sharp pain in every part of her body, so that she suffers torture from it. The oddest feature in her case is perhaps this, that though a Protestant, she says none but a Roman Catholic clergyman can release her from the spirit. We meet strange things in the practice of physic; facts stranger than fiction: but few things in physic are stranger than the phantoms of the crazed mind. There was formerly (now many years ago) an official register kept by the house-surgeon of this infirmary, of the illusions and airy visions of the inmates of our vesanial wards, and it contained some curious matter. Witchcraft frequently recurred in it, but of course amongst various other dreams. In the very same ward in which the former woman was located, I met the following examples, amongst others, within a few years, while it was a male ward. One day, on entering the men's vesanial, I saw a patient sitting up in his bed, covered over with the bed clothes; he was moaning sadly. I asked what was the matter, and he whined out that he had wrapped himself up because of the cold; for the angels he said were pouring water on him down from the ceiling; and he added, he thought it very unkind of them, as he would not serve them in such a manner. In the same ward, not long after, was a man (a quondam campaigner under the Duke in the Peninsula) who, after being some time in the house, had become comparatively tranquil, from a state of cerebral excitement and even dangerous violence. His thoughts always savoured of the magnificent. He took a fancy to me, and promised to make a man of me. He told me he was going to take a tour on the continent, with the royal family in his suite, and he intended to appoint me travelling physician to the party, at a salary of 10,000*l*. per annum. Another day he told me he had a famous plan for the poor, about whose wants he often spoke with apparent feeling. He said he intended soon to put gunpowder under all the towns in England, and blow them up, to make work for the poor in rebuilding them. I do not mention these things, for the idle purpose of raising a titter. My object is very different, namely to excite curiosity in you respecting the inmates of our vesanial wards. I can assure you, that amongst the ten or twelve, or more, persons commonly, for a longer or shorter period, under treatment in that part of the infirmary, you will often meet with facts of high interest. I now draw your attention to them in this pointed way, because they are, so far as I have seen, less attended to by our pupils than other wards, and less, I think, than they deserve. Those wards will show you that, as in the two cases just alluded to, so in most cases, if not all, madness, in whatever form and degree, involves some cerebral disturbance more or less amenable to medicine and professional care. You will see in them the effects of bleeding, antimony, mercury, stimuli, opium, cold applications, &c., administered on the same general principles as in other diseases, though with important differences as to degree of activity, mode of combination, and other details. It is very much during the acuter states, when medicine has most direct power, that our insane patients are admitted, being passed to various asylums, when confirmed or chronic. If you are ever called before a court of inquiry respecting lunacy, &c., you will feel the advantage of having familiarised yourselves with the signs and effects, physical and moral, of mental derangement. Let me add one more reason or inducement to watch the patients in these wards. It is this :—In all ages medical men have been large contributors to the progress of science; every department of human thought and research is indebted more or less to medical learning and talent; and none more than mental science, for which perhaps more has been done by medical men than by any other class of men whatsoever. Not to go too far a field, I may refer to our great reformer Locke as a physician. His system has gone to pieces now some time since, and out of the fragments have been constructed, as a French philosopher (Baron Degerando) has clearly shown, some seven or eight different jarring systems or sub-systems. But the business of reform in mental science has been resumed on other and sounder principles, and by a physician. I mean Dr. Gall ; and phrenology, or the science of mind, when it shall have been disencumbered of numerous crudities, heaped on it by its founder for the most part, will, I make no doubt, generally be regarded as the only system before the public that makes any tolerable approach to what the enlightened common sense of mankind can recognize as real in science, or useful for practical purposes. Now it was to the study of insanity very much that gave Gall the clue; mad people are unconscious witnesses against, and telling illustrations of, the unsoundness of the earlier systems. But I have said more than enough on the point, and must conclude with this, which, if you will, you may consider an apology for alluding to such things as philosophy and mental science in this place of sickness and suffering, or for a moment turning your attention away from practical medicine,—and it is this: Having been for many a long year a physician and practitioner before I had been able practically to study insanity, owing to the exclusion of lunatics, &c., from our hospitals, and of medical students from our lunatic establishments, I have personally experienced the want of that familiarity with mental disease against which I now warn you in time, to provide yourselves, as to a considerable extent you may, in a moderate period in these wards.—*Lond. Lancet.* March 12th, 1842.

